# Cyclodextrins and Their Polymers Affect the Lipid Membrane Permeability and Increase Levofloxacin’s Antibacterial Activity In Vitro

**DOI:** 10.3390/polym14214476

**Published:** 2022-10-22

**Authors:** Anna A. Skuredina, Anastasia S. Tychinina, Irina M. Le-Deygen, Sergey A. Golyshev, Tatiana Yu. Kopnova, Nikolay T. Le, Natalya G. Belogurova, Elena V. Kudryashova

**Affiliations:** 1 Chemistry Department, Lomonosov MSU, 119991 Moscow, Russia; 2 Belozersky Institute of Physico-Chemical Biology, Lomonosov MSU, 119991 Moscow, Russia; 3 Faculty of Physics, Lomonosov MSU, 119991 Moscow, Russia

**Keywords:** cyclodextrin, levofloxacin, liposome, spectroscopy, antibacterial activity

## Abstract

Cyclodextrins (CDs) are promising drug carriers that are used in medicine. We chose CDs with different substituents (polar/apolar, charged/neutral) to obtain polymers (CDpols) with different properties. CDpols are urethanes with average Mw of ~120 kDa; they form nanoparticles 100–150 nm in diameter with variable ζ-potential. We studied the interaction of CD and CDpols with model (liposomal) and bacterial membranes. Both types of CD carriers cause an increase in the liposomal membrane permeability, and for polymers, this effect was almost two times stronger. The formation of CD/CDpols complexes with levofloxacin (LV) enhances LV’s antibacterial action 2-fold in vitro on five bacterial strains. The most pronounced effect was determined for LV-CD complexes. LV-CDs and LV-CDpols adsorb on bacteria, and cell morphology influences this process dramatically. According to TEM studies, the rough surface and proteinaceous fimbria of Gram-negative *E. coli* facilitate the adsorption of CD particles, whereas the smooth surface of Gram-positive bacteria impedes it. In comparison with LV-CDs, LV-CDpols are adsorbed 15% more effectively by *E. coli*, 2.3-fold better by lactobacilli and 5-fold better in the case of *B. subtilis*. CDs and CDpols are not toxic for bacterial cells, but may cause mild defects that, in addition to LV-CD carrier adsorption, improve LV’s antibacterial properties.

## 1. Introduction

Many infectious diseases require complex, long-term medication regimens. Due to the low efficacy and poor bioavailability of some drugs, the standard course of treatment may require high-dosage therapy, potentially causing dangerous side effects [1,2]. Thus, the development of new drug formulations using drug delivery systems with increased bioavailability and safety is of great benefit to society.

FDA approved oligosaccharides, i.e., cyclodextrins (CDs), to design new drug delivery systems. CDs are low cost, possess high solubility and can be administrated in various ways [3]. The modification of CD’s hydroxyl groups by amination, alkylation, etc., widens the perspective of CD usage, and nowadays, not the native CDs but their derivatives are in the spotlight [4]. CDs consist of D-glucopyranose units that form a truncated torus with a hydrophilic surface and a hydrophobic cavity. The hydrophobic cavity enables CDs to encapsulate hydrophobic drug molecules (or their fragments), forming non-covalent inclusion guest–host complexes. Complex formation leads to the improvement of the drug’s physico-chemical properties, namely the increase in its solubility, stability and bioavailability [5,6,7,8].

The modern trend in CD chemistry is the synthesis of polymers based on CD derivatives (CDpols) by various linkers: epichlorohydrin [9,10], diisocyanate [11,12], carbonyldiimidazole [13], etc. CDpols have a clear advantage: cargo molecules form a complex with the CD torus, and, in addition, “get stuck” in a polymer’s network. This makes CDpol carriers promising as drug delivery systems with sustained drug release [12,14].

Commonly, researchers study the physico-chemical properties of drug–CD or drug–CDpol complexes [15,16]; however, the interaction of such systems with biological structures—for instance, membranes—remains under consideration. It is known that CDs do not penetrate through biological barriers, but can adhere to the cell’s surface [17]. Nevertheless, how can one predict CD-based carrier’s interaction with membranes? Given the large number of available CDs with different substituents, it is important to determine the most promising carriers for drug delivery. Recently, we demonstrated that CDs cause mild defects in the liposomal membrane [18], but it is not clear how significant these in vitro-observed defects are in terms of a drug’s antibacterial activity.

Here, we study the interaction between CD-based drug carriers (CDs and CDpols) with liposomal and bacterial membranes in order to establish the effect of CDs on the antibacterial activity of drugs in vitro and the mechanism of such. The purpose of this work is to establish the most promising carrier and determine the specific parameters of bacterial membranes that must be taken into account while designing novel CD-based drug delivery systems.

## 2. Materials and Methods

### 2.1. Materials

2-hydroxypropyl β-cyclodextrin (HPCD), methyl β-cyclodextrin (MCD), levofloxacin (LV), phenolphthalein (PP), Triton X-100, 1,6-hexamethylenediisocyanate and dimethyl sulfoxide (DMSO) were all from Sigma-Aldrich (St. Louis, MO, USA). Sulfobutyl ether β-cyclodextrin sodium salt (SBECD) was from Zibo Qianhui Biotechnology Co. (Zibo, China). Amino β-cyclodextrin (AMCD) was purchased from Shandong Binzhou Zhiyuan (Binzhou, China). Dipalmitoylphosphatidylcholine (DPPL) and cardiolipin (CL; 1,3-bis(sn-3′-phosphatidyl)-sn-glycerin) were purchased from Avanti Polar Lipids (Alabaster, AL, USA). HCl, copper (II) sulfate, ethanol and acetone were all purchased from Reakhim (Russian Federation). Sodium phosphate buffer tablets for solution preparation were obtained from Pan-Eco (Russian Federation). Lead citrate was from Serva (Munich, Germany). Sodium cacodylate, osmium tetroxide and phosphor-tungstic acid were all purchased from SPI (New York, NY, USA).

Gram-negative bacterial strains were *Escherichia coli* MH1 [19] M. Hall, USA), *E. coli* JM109 [20] (J. Messing, USA) and *E. coli* ATCC 25922, and Gram-positive bacterial strains were *Bacillus subtilis* ATCC 6633 (from the Russian Collection of Industrial Microorganisms National Research Institute, Kurchatov Institute) and *Lactobacillus fermentum* 90T-C4 (from Microgen, Pushino, Russian Federation).

### 2.2. CDpol Synthesis

The synthesis of CDpols was performed according to [21]. Briefly, a warm aqueous solution of 5–7 mg/mL HPCD, MCD, SBECD or AMCD was mixed with DMSO for 5 min. The linker’s solution (16.8 mg/mL of 1,6-hexamethelendiisocyanate in DMSO) was added dropwise upon intense stirring. The CD derivative:linker molar ratio was 1:3. The final solution (H_2_O:DMSO ratio was 1:1 vol/vol) was exposed for 3 h at 25 °C. The purification of 5 mL of each sample from organic solvent was conducted by dialysis (MWCO Serva 3.5 kDa membrane) for 24 h at 37 °C with intense shaking and periodic renewal of the external solution (500–600 mL of distilled water). The aqueous solutions were frozen at −18 °C for 12 h, then stored at −70 °C for 24 h and lyophilized for 72 h at −60 °C by a freeze-dryer (Thermo Scientific, Waltham, MA, USA). The concentration of CD-torus in CDpols was determined by FTIR spectroscopy in accordance with the intensity of the band in the region 1050–1030 cm^−1^ corresponding to the C-O-C bond of CDs.

### 2.3. Preparation of Liposomes Loaded with Phenolphthalein

Briefly, 25 mg/mL of DPPC and 25 mg/mL of CL lipids (both in chloroform) were mixed to obtain a solution with a DPPC:CL ratio of 80:20 (weight %). The removal of chloroform was conducted by evaporation at 50–55 °C using a vacuum rotary evaporator. The obtained thin lipid film in a glass flask was dispersed in 10^−6^ M phenolphthalein solution (0.02 M sodium phosphate buffer solution, pH 7.4). The liposomes (3 mg/mL of lipids) were sonicated at a frequency of 22 kHz three times for 200 s each using a homogenizer (4710 model, Cole-Parmer Instrument, Vernon Hills, IL, USA) at 50–55 °C. Unloaded phenolphthalein was removed by dialysis (MWCO Serva 3.5 kDa membrane) for 3 h at 25 °C with intense shaking and periodic renewal of the external solution (500–600 mL 0.02 M sodium phosphate buffer solution, pH 7.4).

### 2.4. The Release Kinetics of Phenolphthalein from the Liposomes

Briefly, 2 mL of liposome solution (3 mg/mL of lipids, DPPC:CL = 80:20 (weight%)) loaded with phenolphthalein was transferred to an Orange Scientific dialysis bag (MWCO 3.5 kDa), which was placed in 2 mL of 0.02 M sodium borate buffer (pH 10.7). Solutions of all CDs, CDpols (C_CD torus_ in final solution was 30 mg/mL) or Triton X-100 were added to the liposome solution by adding to the dialysis bag. The systems were stirred for 1.5 h at 37 °C. UV spectra of the external solution were recorded every 10–15 min.

### 2.5. Preparation of LV-CD and LV-CDpol Complexes

CDs and CDpols were dissolved in 0.1 mM HCl pH 4.0. LV solution in the same pH was added to the CD carrier solution to obtain a molar ratio of CD torus:LV of 1:1. The complexes were stirred for 1 h at 37 °C. To study the interaction of LV-CD or LV-CDpol with the liposomes or bacterial cells, the prepared complexes were diluted with 0.02 M sodium phosphate buffer solution (pH 7.4) immediately before the experiment.

### 2.6. Nanoparticle Tracking Analysis (NTA)

NTA was performed using the Nanosight LM10-HS instrument (Nanosight Ltd., Malvern, UK). The CDpol samples were diluted by Milli-Q purified water to obtain the solutions with ~10^8^ particles/mL. The measurements were carried out three times for each sample, and the values are reported with standard deviations.

### 2.7. Molecular Weight of CDpols

We determined CDpols’ Mr by the following formula:

$$\mathrm{Mr}=\frac{\left[CD\right]\times N_{A}}{n}\times Mr_{m}$$

The number of the particles *n* (particles/mL) was determined by NTA [22]. Other variables are the concentration of CD torus [*CD*] (mole/mL) in accordance with dilution, Avogadro constant *N_A_* and molecular weight of the repeating unit (CD and linker) *Mr_m_*.

### 2.8. Dynamic Light Scattering (DLS)

DLS was used to determine ζ-potentials of the samples (Zetasizer Nano S, Malvern, with 4 mW He–Ne-laser, 633 nm; Malvern, UK). The experiments were performed at 25 °C using the correlation of the K7032-09 Correlator System (Malvern, UK) and Zetasizer software. The measurements were carried out three times for each sample, and the values are reported with standard deviations.

### 2.9. UV Spectroscopy

UV spectroscopy was conducted using Ultrospec 2100 pro instrument (Amersham Biosciences, Amersharm, UK). The spectra of released phenolphthalein were recorded within a wavelength range from 400 to 650 nm in a 1 mL quartz cell (Hellma Analytics). The concentration of phenolphthalein was determined at a wavelength of 550 nm.

### 2.10. NMR Spectroscopy

^1^H NMR spectroscopy was conducted using Bruker Avance 400 spectrometer (Reinshtetten, Germany). First, 5–10 mg of the sample was dissolved in D_2_O, and ^1^H NMR spectra were recorded with operating frequencies of 400 MHz. Chemical shifts (*δ*) in ppm are reported as quoted relative to the residual signals of D_2_O (4.79 ppm).

### 2.11. Fluorescence Spectroscopy

Emission spectra of LV were recorded using the Varian Cary Eclipse Fluorimeter (United States). The measurements were carried out at λ_ex_ = 289 nm within a wavelength range of 400–550 nm. LV demonstrates a peak with a maximum at 456 nm.

### 2.12. FTIR Spectroscopy

FTIR spectroscopy was conducted using a Tensor 27 instrument (Bruker, Germany) equipped with a liquid nitrogen-cooled MCT detector, a thermostat (Huber, Offenburg, Germany), an attenuated total reflection cell (Bruker, Ettlingen, Germany) and a ZnSe single-reflection crystal. The spectra of the samples (40–50 μL) were recorded three times (70 scans each time) within a range of 3000–900 cm^−1^ at a resolution of 1 cm^−1^ at 22 °C. Dry air was pumped through the system by the air compressor (Jun-Air, Munich, Germany). The background (buffer solution) was recorded in the same manner. The spectra were analyzed using the Opus 7.0 software.

### 2.13. Powder X-ray Diffraction (PXRD) Analysis

PXRD patterns of 5 mg of LV or its complexes with CD carriers were recorded on a Rigaku SmartLab (Tokio, Japan)-equipped copper X-ray anode tube. The scanning range was 1.5–80.0° with a step size of 5° per second. X-rays were generated with settings 60 kV and 1.5 kW.

### 2.14. In Vitro Experiments Using Escherichia coli, Lactobacillus Fermentum and Bacillus subtilis Bacterial Strains

We used the overnight culture (the bacteria were cultured in Luria Bertuni (LB) liquid medium for 12 h, except *Lactobacillus fermentum* 90T-C4, for which De Man, Rogosa and Sharpe agar was used). The determination of minimum inhibition (MIC) concentration was performed in solid media by agar the well diffusion method [23]. Briefly, 500 μL of overnight cell culture was distributed over the surface of agar on Petri dishes. Then, 6 wells 9 mm in diameter were incised in the medium by a sterile plastic pipette tip. Then, 50 μL of the samples (LV concentration was 0.01–0.5 μg/mL for all *E. coli* strains and *B. subtilis* ATCC 6633; 20–100 μg/mL for *L. fermentum* 90T-C4) was put into the wells. In 20 min, the plates were placed into the incubator at 37 °C. After 24 h, the appeared inhibition zones were analyzed. The experiments were carried out three times for each sample, and MIC values are reported with standard deviations.

For the ultrastructural studies of bacteria, the pelleted cells (900 μL of the overnight culture) were fixed with 2.5% glutaraldehyde solution (100 mM sodium cacodylate) for 24 h. Then, bacterial pellets were rinsed three times for 5 min in 100 mM sodium cacodylate under slight agitation and were post-fixed with 1% OsO_4_ (100 mM sodium cacodylate) for 1 h at +4 °C. The fixed cells were dehydrated by increasing ethanol concentrations (50%–70%–96%). The ethanol was replaced by acetone, followed by epoxy resin–acetone mixtures with increasing resin content. After replacing the mixture with pure resin (Spi-pon 812, SPI Supplies), the samples were exposed at +70 °C for 48 h. Ultrathin sections with a nominal thickness of 80 nm were prepared using Reichert-Jung Ultracut E ultramicrotome equipped with a Diatome Ultra 45 diamond knife. The sections were mounted on the formvar-coated copper slot-grids, and post-stained with lead citrate for 3 min.

For the study of bacterial surface morphology, negative staining was conducted. First, 15 μL of the bacterial suspension in water was placed on the formvar-coated 200-mesh copper grids for 60–90 s. The drops were blotted with the filter paper. Then, 10 μL of 1% aqueous phosphotungstic acid was applied onto the grids for 20 s and blotted with the filter paper. Finally, the stained samples were air-dried.

For TEM micrograph of CDpol adsorption on *B. subtilis*, we preincubated 5 mg/mL HPCDpol with 0.1 M copper (II) sulfate solution for 1 h. We centrifuged the sample (Eppendorf Minispin, 10,000 rpm/min, 10 min) and removed unbonded copper (II) sulfate solution. HPCDpol-Cu^2+^ was washed 3 times using Milli-Q purified water. Next, 900 μL of overnight *B. subtilis *was centrifuged (Eppendorf Minispin, 7000 rpm/min, 5 min) and washed 3 times with Milli-Q purified water. Then, the HPCDpol-Cu2+ was added to bacteria cells. After 20 min of incubation, 25 μL of the sample was placed on the copper grids for 40–60 s. The drops were blotted with the filter paper, and the sample was dried in air.

The samples then were analyzed and imaged on a JEM-1400 electron microscope (Jeol, Tokio, Japan) running at 80 kV and equipped with a Quemesa digital camera (OSIS, Münster, Germany).

### 2.15. The LV-CD and LV-CDpol Adsorption on the Cells

The adsorption curves were obtained using the overnight bacteria culture (~8×10^8^ colony-forming units (CFU)/mL). A total of 900 μL of bacteria was centrifuged (Eppendorf Minispin, 7000 rpm/min, 5 min) and washed 3 times with sterile 0.02 M Na-phosphate buffer solution buffer (pH 7.4). The samples containing different amounts of LV-CD or LV-CDpols in sterile buffer (pH 7.4) were added to the bacteria pellets and mixed properly. After 1 h of incubation (37 °C, 100 rpm), the samples were centrifuged (7000 rpm/min, 5 min), and the emission spectra of supernatant were recorded.

## 3. Results and Discussion

**Figure 1:**
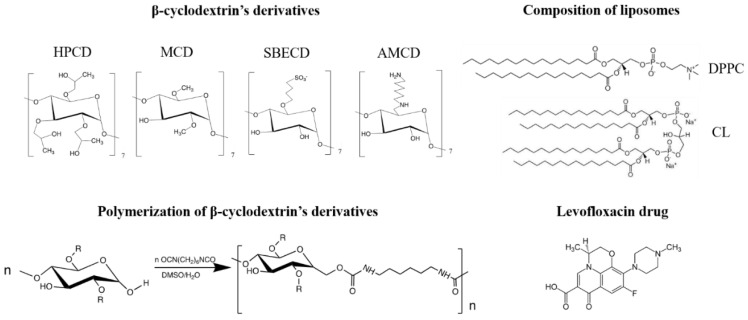
The structure of the β-cyclodextrin derivatives and their polymers, lipids used for liposome production and levofloxacin.

For the in-depth study of CD and CDpol interaction mechanisms with liposomal and cell membranes, we chose oligosaccharides with different substituents (Figure 1): 2-hydroxypropyl-β-cyclodextrin with a polar uncharged substituent (HPCD), methyl-β-cyclodextrin with a nonpolar uncharged substituent (MCD), negatively charged sulfobutyl ether β-cyclodextrin (SBECD) and positively charged amino-β-cyclodextrin (AMCD). We selected well-known fluoroquinolone levofloxacin (LV) as a model drug molecule suitable for guest–host complex formation with CD [6]. LV is widely used in antibacterial therapy of various infections, including COVID-19-associated pneumonia [24,25].

We synthesized CDpols using 1,6-hexamethelenediisocyanate at a low molar excess of crosslinking agent to obtain soluble polymers that can be used as a carriers for drug delivery.

### 3.1. Characterization of Physico-Chemical Properties of CDs and CDpols

The size, charge and the structure of CDs and CDpols might affect the interaction between the carrier and the membrane, so we studied the parameters by nanoparticle tracking analysis (NTA) and DLS (Table 1). As expected, HPCD and MCD are uncharged, while SBECD and AMCD demonstrate negative and positive ζ-potential, respectively. Thus, we investigated the influence of CD’s charge on its interaction with the bilayer.

CDs crosslinking with 1,6-hexamethelenediisocyanate leads to the formation of homogeneous nanoparticles with the average molar mass of ~100−130 kDa and hydrodynamic diameter of 100–150 nm. MCDpol demonstrates the highest mass and particle size. Among all CDs, MCDs have the lowest number of available hydroxyl groups that react with the crosslinking agent, so MCDpol might have a linear polymeric network, whereas other CDpols have a more branched polymeric network.

Interestingly, CDpols (except SBECDpol) have pronounced positive ζ-potential. This effect might be explained by spontaneous hydrolysis of 1,6-diisocyanate [27] during CDpol’s synthesis and the formation of positively charged functional groups that contribute to the ζ-potential of the polymers. In the case of SBECD, multiple negatively charged sulfo groups might give a greater contribution that overlaps the positive charge value.

The structure of the polymers was studied by FTIR and NMR spectroscopy (Figure S1). In the ^1^H NMR spectra of CDpols, we observed the peaks of monomer units and also the signals that were correlated with the methylene groups of the attached linker [28,29]. To estimate the number of attached linkers per one CD torus, the spectra were normalized by a signal corresponding to the H1 D-glucopyranose unit (doublet 5.25 ppm and 4.96 ppm); on average, there are two to three spacers per oligosaccharide, i.e., polymers are not tightly crosslinked. It is important to note that in the concentrations required to obtain NMR spectra, CDpols do not completely dissolve in D_2_O, so the obtained values may be slightly underestimated.

In CDpol’s FTIR spectra, we observed the appearance of a 1575 cm^−1^ peak that corresponds to amide bonds in the urethane group. This means that the synthesis was performed in accordance with the scheme in Figure 1. Furthermore, CDpol’s synthesis causes the appearance of shoulders of the absorption bands that correspond to C-O-C in glucopyranose residues (1030–1040 cm^−1^). The changes in the microenvironment of C–O–C bands between glucopyranose residues in CD derivatives after crosslinking might be the reason. The 951 cm^−1^ peaks determined in CDpol’s spectra correspond to the C-N bond in amines. Hydrolysis of isocyanate groups leads to amine formation, which contributes to the positive ζ-potential (Table 1).

Thus, we obtained two groups of samples that possess different sizes (~0.1 nm and 100–150 nm), masses (~1.2–2 kDa and ~120–150 kDa) and surface charges (positive, negative, neutral). These parameters might affect the carrier’s interaction with biological membranes.

### 3.2. Interaction of Liposomes with CDs and CDpols

First, we studied the effect of CD and CDpols on the integrity of the model biological membrane, i.e., the liposomal bilayer (DPPL/CL = 80/20 (weight %)). CDs and CDpols adsorb on liposomes that can be monitored by DLS: the ζ-potential of liposome (−20 mV) slightly increases with surface charge neutralization (Table 1). The CD carrier’s adsorption might lead to the changes in membrane properties.

**Figure 2:**
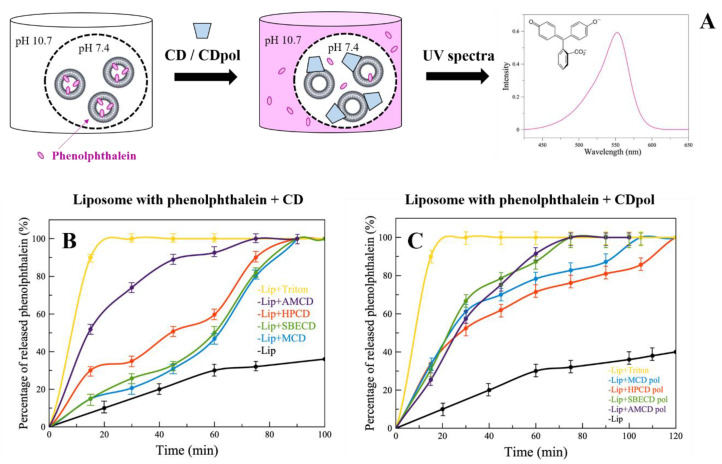
UV spectra of phenolphthalein (**A**), pH 10.7 (0.02 M Na-borate buffer solution), 22 °C. The release kinetics of PP from liposomes DPPC:CL = 80:20 (weight %) after adding CD (**B**) or CDpol (**C**), C_CD torus_ = 30 mg/mL, pH 7.4 (0.02 M Na-phosphate buffer solution), pH 10.7 (0.02 M Na-borate buffer solution), 37 °C.

For instance, CDs cause defects in the liposomal bilayer, but still, it is not clear which CD parameter is crucial for this effect. The occurrence of defects in the liposomal bilayer and violation of the integrity of liposomal membrane are studied by a number of authors via the analysis of the release kinetics of dyes from vesicles [30]. We loaded anionic liposomes (DPPL/CL = 80/20 (weight %)) with indicator phenolphthalein (PP) at pH 7.4 (colorless solution). Liposomes were separated from the external solution by a dialysis membrane. The defects of liposomal membrane cause the PP’s release, which can be detected in the external buffer solution with a pH of 10.7 (pink solution) at a wavelength of 550 nm (Figure 2A).

Control liposomes (in the absence of CD) release about 40% of PP in 120 min (Figure 2B,C, black curves). For the complete destruction of liposomes (100% release of PP), the well-known surfactant Triton X-100 (0.4% by weight) was added. Triton X-100 significantly accelerates PP’s release, and 100% of the indicator is released after 20 min (Figure 2B,C, yellow curves).

When CD derivatives are added, a sustained PP release is observed (Figure 2B) compared to the control, apparently due to CD’s adsorption on the liposomes and the formation of defects in the bilayer. It is important to note that the highest release rate is characteristic of HPCD and AMCD. This is probably due to the fact that HPCD has the largest number of hydroxyl groups in its structure, and consequently, more hydrogen bonds are formed between the phosphate groups of lipids and −OH groups of HPCD. AMCD has a positively charged amino group (Table 1), so electrostatic interactions are expected between the charged –NH_2_ of AMCD and negatively charged lipid heads. This is in agreement with our previous research [31]; we demonstrated by FTIR spectroscopy the interaction between PO_2_^−^ groups of the lipids with -OH and -NH_2_ groups on chitosan and chitosan–mannose conjugate.

Thus, CD’s adsorption on the liposomal membrane plays an important role in the formation of defects in the bilayer. According to the degree of occurrence of defects, we have ranked CDs as follows: AMCD >> HPCD > MCD ~ SBCD.

In the case of CDpols (Figure 2C), the release acceleration is 2 times higher than for CDs during the first 60 min. Most likely, CDpol’s particles have more functional groups for the interaction with the vesicles. Moreover, polymer particles might have a higher charge density that also affects the occurrence of defects. For instance, AMCD and AMCDpol have almost the same effect on membrane integrity due to the close ζ-potential values (Table 1).

Thus, CDpol causes 2 times more defects in the liposomal bilayer than CDs. Such effects can lead to bacterial membrane damage and/or contribute to improving drug penetration through biological barriers, therefore increasing antibacterial activity.

### 3.3. Antibacterial Activity of LV and Its Complexes with CD Derivatives

**Figure 3:**
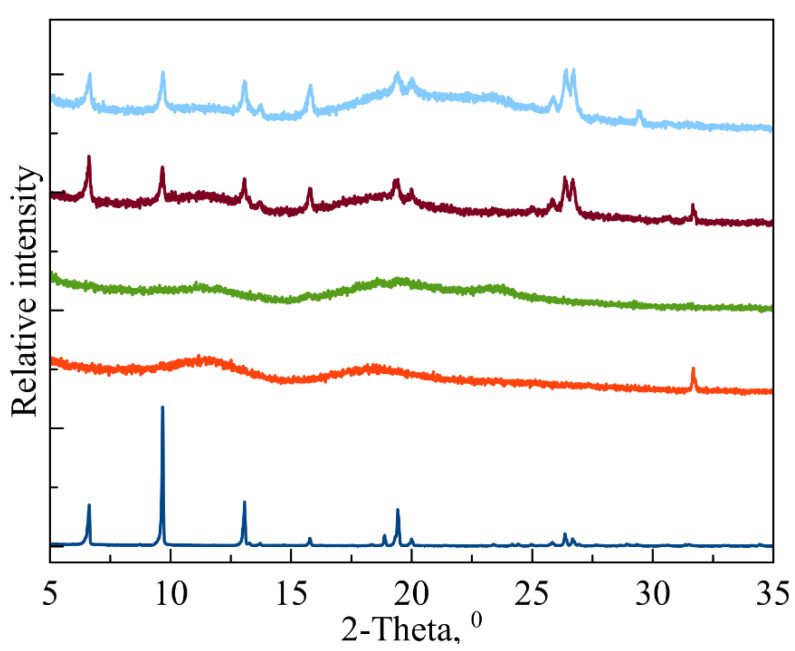
Experimental PXRD patterns for LV (navy blue), MCD (red) and MCDpol (green); LV-MCD (brown) and LV-MCDpol complexes (baby blue). From 35° to 80°, no more signals were observed.

For in vitro studies, we chose fluoroquinolone levofloxacin (LV) as a broad-spectrum antibacterial agent [32] that forms guest–host complexes with CD carriers [33]. First, in order to discover the effectiveness of LV’s incorporation into CD and CDpols in a solid state, we obtained powder X-ray diffraction (PXRD) patterns (Figure 3) as a gold standard procedure described for CD complexes, e.g., in [34]. The PXRD pattern of LV (navy blue line) corresponds to a crystalline LV (CCDC code: YUJNUM02 deposited in Cambridge Structural Database) [35]. The PXRD patterns for MCD (red) and MCDpol (green) are mainly of amorphous phase, which is in agreement with previously published data [36]. When LV is complexed with both CD carriers, the PXRD pattern changes significantly: the degree of crystallinity measured at the integral intensities of the halo and crystalline peaks is around 20%. Previously, we observed this type of change for HPCD complex with another fluoroquinolone moxifloxacin [6]. Thus, in the solid state, the main part of LV is complexed with CD.

We investigated the effect of CD carriers on LV’s properties in vitro on five bacterial strains: Gram-negative *Escherichia coli* MH1, *E. coli* JM109 and *E. coli* ATCC 25922, and Gram-positive *Lactobacillus fermentum* 90T-C4 and *Bacillus subtilis* ATCC 6633. It is known that Gram-negative bacteria have outer lipid membranes, whereas Gram-positive cells have peptidoglycans, so we expected especially pronounced effects on *E. coli* [37].

**Figure 4:**
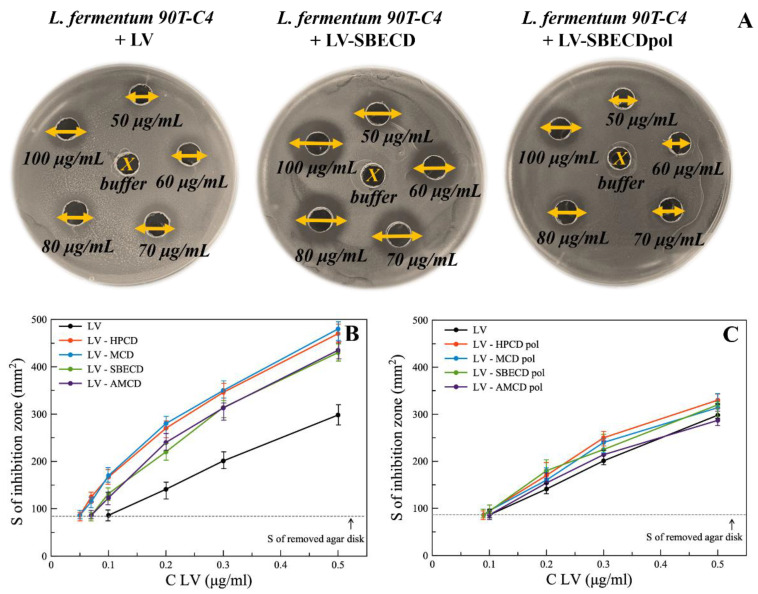
(**A**) The diameters of inhibition zones (mm) on Petri dishes with free LV and its complexes with SBECD and SBECDpol in the concentration range 50–100 μg/mL, agar well diffusion method, *L. fermentum* 90T-C4 pH 7.4 (0.02 M Na-phosphate buffer solution), 37 °C, 24 h of incubation. (**B,C**) The area of inhibition of *E. coli* ATCC 25922 bacterial growth depending on the concentration of the drug, agar well diffusion method, pH 7.4 (0.02 M Na-phosphate buffer solution), 37 °C, 24 h of incubation.

The agar well diffusion method was used to study the antibacterial properties of LV formulations (Figure 4A), being one of the most robust techniques to assess a set of drug concentrations simultaneously [38,39].

Minimum inhibition concentration (MIC) was assumed as LV’s concentration at which the inhibition area of bacteria equals the area of the removed agar disk. Table 2 represents MIC values for all bacterial strains.

MIC values differ significantly not only between bacterial species, but also within bacterial strains. Among *E. coli* strains, the lowest MIC value of 0.1 μg/mL is observed for *E. coli* MH1 and *E. coli* ATCC 25922 and the highest for *E. coli* JM109 (1 μg/mL). Higher resistance of *E. coli* JM109 might be associated with *gyrA96* mutation [20,40], which is the main target of LV [41]. For Gram-positive bacteria, MIC*_L. fermentum_*_90T-C4_ is more than 23 times higher than MIC*_B. subtilis_*, which seems beneficial since lactobacilli are associated with probiotic properties [42].

All studied CDs and CDpols possess no antibacterial effect. This result is in agreement with previous studies of these CD derivatives [12,43]. Nevertheless, the CD substituent plays a key role in the CD’s in vitro properties. In [44], the novel alkylamino CDs were synthesized that differ by the length and branching of terminal alkyl chain. The authors demonstrate CD’s strong antibacterial activity depending on the number of carbon atoms in the alkyl chain (from four to seven). The amino CDs bearing alkyl groups consisting of five to six carbon atoms exhibit the highest antibacterial activity, equivalent to that of natural melittin (antibiotic peptide). If the number of carbon atoms is four, then almost no effect is observed. These results are in agreement with our study, since we used the CD derivatives with small terminal alkyl groups (–CH_3_ with one carbon atom) or terminal polar/charged groups.

LV-CD complexes demonstrate lower MIC values than free LV for both Gram-positive and Gram-negative cells. For *E. coli* MH1, *E. coli* JM109 and *B. subtilis* ATCC 6633, we observed only slight or almost no CD influence on LV’s MIC. However, for *E. coli* ATCC 25922 and *L. fermentum* 90T-C4, CD lowers MIC_LV_ dramatically (almost 2-fold). The increase in the drug’s action and the decrease in its MIC was also observed for CD complexes with essential oils (carvacrol and 2-pentanoylfuran) [45]. This effect might be explained by the increase in the drug’s solubility, adsorption of LV-CD complexes on bacteria, the occurrence of membrane defects and/or the facilitation of LV’s diffusion into the cells. Nevertheless, no specific influence of CD substituent on LV’s activity was detected. All CDs demonstrate similar effects.

MICs _LV-CDpols_ are close or slightly lower than MIC_LV_. A less significant influence on LV’s action for CDpols compared to CD might be explained by the fact that large particles have limited diffusion through agar media; therefore, the antibacterial activity is comparable to the bacterial activity of LV.

Hence, the CDs and CDpols increase LV’s antibacterial activity on Gram-positive and Gram-negative bacteria. For understanding the mechanism of such effects, we investigated the cells’ state during LV carrier exposure.

### 3.4. Influence of CDs and CDpols on Bacterial Features

**Figure 5:**
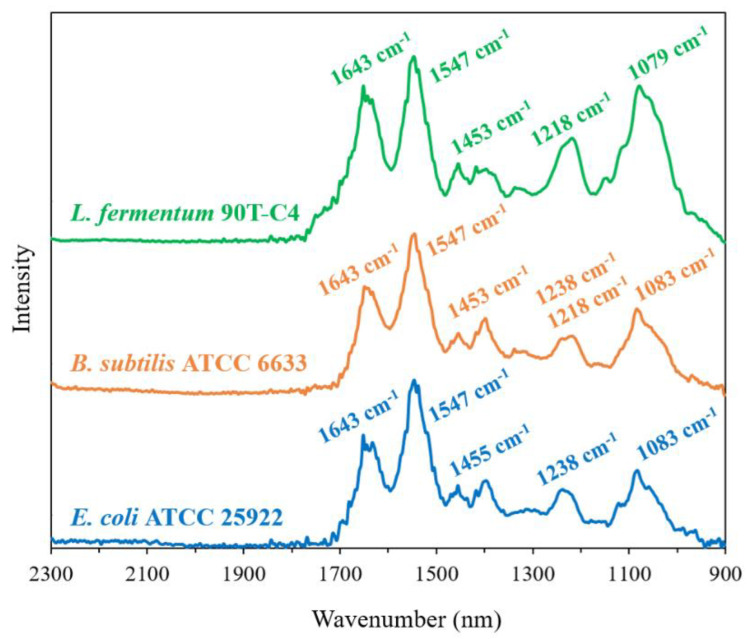
FTIR spectra of *L. fermentum* 90T-C4, *B. subtilis* ATCC6633 and *E. coli* ATCC 25922 in the range 2300–900 cm^−1^ of pure cultures of bacteria, ~10^11^ CFU/mL, H_2_O, 22 °C.

First, we studied the cells’ composition by FTIR, a powerful tool for the differentiation and identification of bacteria. The FTIR spectra of cells (Figure 5) have a number of distinctive peaks that are typical for all bacterial species [46]. The considerable region is 1300–900 cm^−1^, which contains signals from several cell components, including DNA and phospholipids, as well as strong absorbance bands from complex sugar modes. Significant intense bands within this region have been reported for different bacteria and assigned to the cell wall components [47,48].

The three studied Gram-negative *E. coli* strains demonstrate the same position and close intensity of the peaks. For *L. fermentum* 90T-C4, 1218 cm^−1^ and 1079 cm^−1^ bands are remarkably intense compared to *E. coli.* This finding was expected since these peaks are correlated with polysaccharides that are contained to a large extent in Gram-positive bacteria’s cell wall [46]. Interestingly, for Gram-positive *B. subtilis* ATCC6633 and Gram-negative *E. coli*, we observed similar characteristics of its FTIR spectra that might be related to the special features of *B. subtilis.* Similar compositions of *E. coli* and *B. subtilis* might lead to the close MIC values.

We recently demonstrated that FTIR spectroscopy is a powerful tool to study the interaction between HPCD and liposomal membrane [18]. Briefly, HPCD caused the changes in the position of the bands that corresponded to CH_2_, PO_2_^−^ and C=O groups in the FTIR spectra of liposomes. Thus, we expected to observe the changes in bacteria’s FTIR spectra that can appear due to CD administration, mainly the 1300–900 cm^−1^ region that corresponds to the cell’s surface. However, interactions of CDs or CDpols with bacteria did not lead to the changes in cell spectra (C_CD torus_ ~500 μg/mL). We suppose that, unlike liposomes, where lipids are more accessible for interaction with CD carriers, bacterial membranes have a number of proteins and the interaction is more complicated, or the carriers might have a mild effect on bacteria that cannot be studied by FTIR spectroscopy. Thus, we conducted the ultrastructural studies by transmission electron microscopy (TEM).

TEM is an efficient tool to examine living, dead and dividing cells [12]. What is more interesting in our case is that one can detect the changes in bacteria’s structure and determine the mechanism of substance action. For instance, chitosan damages the bacterial membrane [49]; peptide nisin causes partial cell wall detachment from the plasma membrane [50] and leaking cell contents [51]; andrographolide derivative leads to the formation of large cytoplasmic aggregates, cell elongation with abnormal cell septation, cytoplasmic disintegration and finally, cell lysis [52].

**Figure 6:**
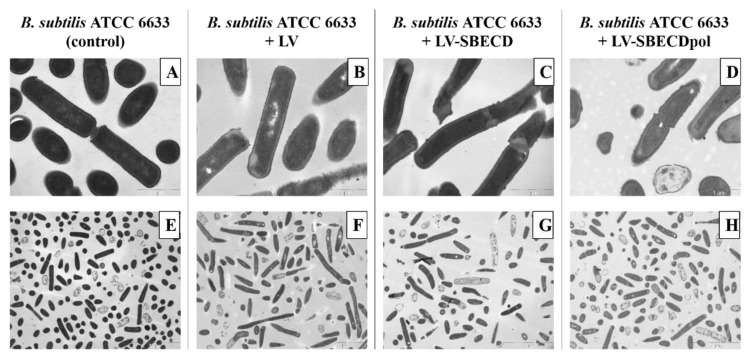
The TEM micrographs of *B. subtilis* ATCC6633: overnight culture, cells incubated with LV, LV-SBECD or LV-SBECDpol for 24 h at 37 °C. C_LV_ = 2.5 μg/mL. Scale bar: 1 μm (**A**–**D**) and 5 μm (**E**–**H**).

The control *B. subtilis* ATCC6633′s ultrastructure demonstrates a homogeneous bacteria state: all cells are in the same growth phase (Figure 6A,E). On the micrographs of bacteria exposed to LV, LV–SBECD or LV–SBECDpol for 24 h (Figure 6B–D,F–H), the total number of cells is lower than in the control culture. Bacteria demonstrates morphological heterogeneity and many dead cells were detected; however, the majority of the bacterial cells retained a typical structural feature.

Still, no specific effect of CD or CDpols on the morphology the cell wall, membrane or cytoplasm was detected that proves that CDs might only have a mild effect on the cell membrane. The defects that may occur are not lethal for cells, so there must be other reasons for the 2-fold increase in LV’s activity in vitro. One of the main factors might be associated with the carrier’s adsorption on the bacterial surface, resulting in a high local concentration of the drug.

### 3.5. Adsorption of LV-CDs and LV-CDpols on Bacteria

As shown in the previous section, electrostatic interaction between the liposomal membrane and CD-based carrier is one of the main factors of their interaction. Thus, we studied the ζ-potential of the bacterial surface (Table 3).

All bacteria possess pronounced negative ζ-potential that is in agreement with literature data [37,53]. Gram-positive bacteria demonstrate a higher charge than Gram-negative, so we expected better LV-CD and LV-CDpol adsorption on *L. fermentum* 90T-C4 and *B. subtilis* ATCC6633. Nevertheless, cells and liposomes have comparable ζ-potential values, and we expected to determine similar tendencies of CD’s interaction with the bacterial membrane, which were observed when studying CD liposomes.

**Figure 7:**
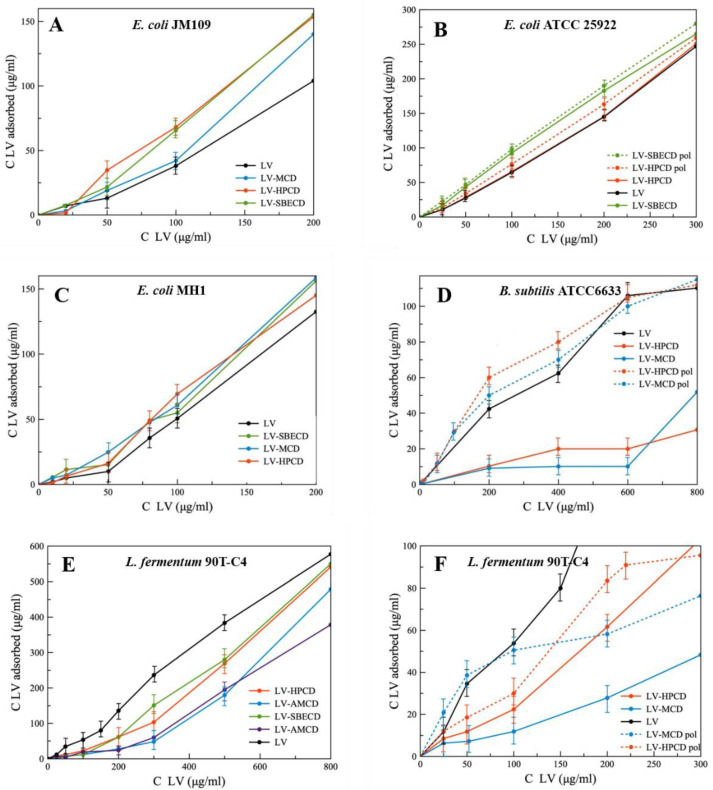
The absorption curves of LV, LV–CD and LV-CDpol on bacterial strains, ~1 × 10^9^ cells/mL, pH 7.4 (0.02 M Na-phosphate buffer solution), 1 h of incubation, 37 °C. (**A**) *E. coli* JM109; (**B**) *E. coli* ATCC 25922; (**C**) *E. coli* MH1; (**D**) *B. subtilis* ATCC6633; (**E**) *Lactobacillus fermentum* 90T-C4; (**F**) *Lactobacillus fermentum* 90T-C4.

The adsorption curves are presented as the dependence of absorbed LV on the added LV concentration (Figure 7). Comparing the slopes of linear curves, we found that for Gram-negative strains, the formation of LV–CD complexes leads to the increase in LV’s absorption, whereas for Gram-positive bacteria, the opposite trend is observed. This unexpected result indicates the importance of bacterial membranes in the interaction with CD carriers.

On average, among all LV-CD complexes, HPCD demonstrates the most pronounced increase in LV’s adsorption on bacteria. As HPCD demonstrated high acceleration of PP’s release from liposomes, we consider that electrostatic interactions are not the main factor in CD’s adsorption on bacterial cells. The mechanism of interaction between CDs and multi-component bacterial membrane must be studied in detail.

For all bacterial strains, LV-CDpols are adsorbed better than LV-CDs, which confirms the results obtained during the experiment with PP’s release from liposomes (Figure 2). On average, polymers are adsorbed 15% more effectively for *E. coli*, in 2.3-fold for lactobacilli and 5-fold for *B. subtilis*. That indicates LV-CDpols are more promising drug carriers than LV-CD.

The most significant effect on LV’s adsorption is observed for HPCDpol and MCDpol which possess the highest ζ-potential (Table 1). Thus, for CDpols, electrostatic interactions play a key role in CDpol–cell membrane interactions. We suppose that multiple charged functional groups help CDpols to be retained at many binding sites on cells.

The differences between the morphology of bacterial surface might also influence LV-CDs and LV-CDpols adsorption, so we studied the bacterial surface by TEM.

### 3.6. The Influence of Bacterial Cell’s Morphology on LV Adsorption

**Figure 8:**
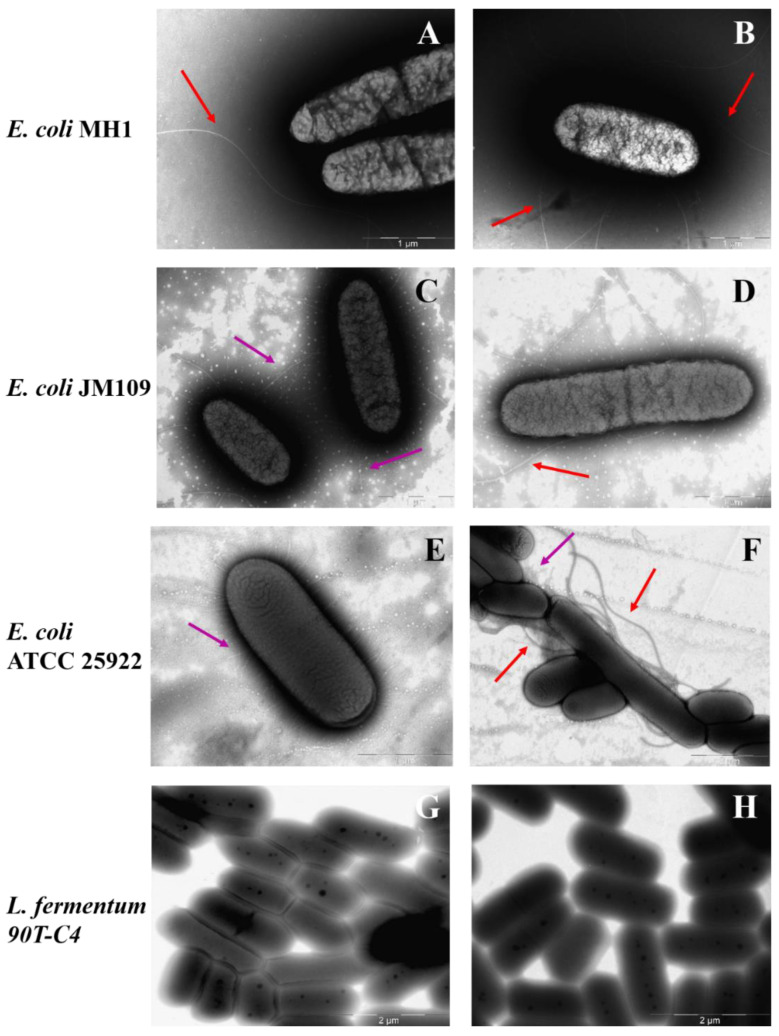
The morphology of bacterial surface: *E. coli* MH1 (**A**,**B**), *E. coli* JM109 (**C**,**D**), *E. coli* ATCC 25922 (**E**,**F**), *L. fermentum* 90T-C4 (**G**,**H**). Purple arrows indicate fimbria and red arrows indicate flagella. Scale bars: 1 μm (**A**–**E**) and 2 μm (**F**–**H**).

Figure 8 demonstrates TEM micrographs of bacterial strains. All Gram-negative *E. coli* strains possess a number of long (approximately 5 μm) flagella, more than one for each cell. In addition, the surface of *E. coli* JM109 and *E. coli* ATCC 25922 is covered with proteinaceous fimbria that are 300–1000 nm in length. Visually, *E. coli* ATCC 25922 is characterized by more numerous fimbriae that are thinner and shorter compared to the *E. coli* JM109. Fimbria mainly help the bacteria to adhere and transfer genetic information [54] that might improve the adhesion of LV’s forms. Indeed, for *E. coli* JM109 and *E. coli* ATCC 25922, the adsorption is in almost 2 times higher than for *E. coli* MH1 (Table S1).

In contrast, Gram-positive *L. fermentum* 90T-C4 do not have flagella or fimbriae. The cell surface is smooth and homogeneous, while *E. coli* is characterized by a rough surface. The micrograph of lactobacilli also clearly shows the thick cell wall of peptidoglycan. *B. subtilis* ATCC6633 demonstrated low adhesion ability on the formvar-coated EM grids that might be due to lipophilic bacteria capsule. The micrographs uncovered only numerous flagella (data not shown).

The smooth surface of Gram-positive bacteria might be the reason for the lower adhesion on the cells, whereas the rough surface of Gram-negative bacteria with fimbria increases the adhesion of both LV-CD and LV-CDpols, demonstrated in the previous section (Figure 7).

### 3.7. Visualization of CDpol Adsorption on Bacteria

To confirm CDpol adsorption on bacterial cells, we visualized the adhesion by TEM. Due to high solubility and a lack of heavy atoms, CD’s visualization by TEM is complicated. Some authors obtain metal complexes [55] or use drugs with an aromatic core with additional contrast by sodium phosphotungstate [56]. We obtained HPCDpol-Cu^2+^ using copper (II) sulfate. However, even contrasted polymer was challenging to visualize.

In order to avoid the desorption of particles from the cell surface, no phosphotungstic acid was used. In such conditions, bacteria appear as black oval shapes with light gray areas. No flagella or fimbria are visible. Earlier, Nadtochenko et al. demonstrated similar bacteria images [57].

**Figure 9:**
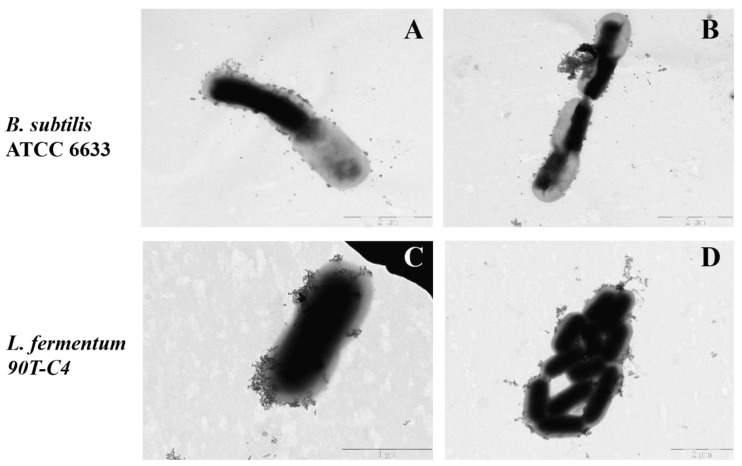
The TEM microphotographs of HPCDpol-Cu^2+^ particle adsorption on *B. subtilis* ATCC 6633 (**A**,**B**) and *L. fermentum* 90T-C4 (**C**,**D**), H_2_O, 20 min of incubation, 37 °C, C(Cu^2+^) ~ 0.001 M, 80 mV. Scale bars: 1 μm (**C**) and 2 μm (**A**,**B**,**D**).

It should be noted that cell images vary significantly within bacterial species. We selected the most suitable copper (II) ion concentration that does not lead to observable changes in the bacteria’s shape or cytoplasm. Figure 9 demonstrates particle absorption on *B. subtilis* ATCC 6633 and *L. fermentum* 90T-C4.

According to the results, HPCDpol-Cu^2+^ forms aggregates, but does not possess any selectivity to the bacterial surface—the particles distribute evenly over the cell’s surface. No visible defects in bacterial surface or cell shape are observed, which proves that CDpols have a mild effect on bacteria. Besides the defects in the cell surface, CD and CDpol adsorption on bacteria lead to high local concentrations of LV that consequently lower MIC_LV_ by almost 2 times. Thus, the formation of CD–drug complexes is of great interest to develop drug formulations with increased antibacterial action.

## 4. Conclusions

Here, we studied the interaction of biomembranes (both liposomal and bacterial) with drug carriers CDs and CDpols that differ in size, mass and ζ-potential (Table 1). The crosslinking of CDs with 1.6-hexamethelenediisocyanate led to the formation of the urethane nanoparticle with terminally charged NH_2_ groups, which form due to the hydrolysis of isocyanine groups. We found that CD and CDpols adsorb on the liposomal bilayer (DPPC/CL = 80/20 (weight %)) and cause defects. Pronounced positive ζ-potential (>10 mV), multiple -OH groups and nanoparticle structure increase the occurrence of defects by 2 times. In the case of the bacterial membrane, the interaction is more complicated.

PXRD experiments showed that the main part of the drug LV is complexed with the CD matrix. For LV-CD’s adsorption on bacteria, electrostatic interactions are not the main factor, whereas for LV-CDpols, adsorption is associated with multiple electrostatic interactions between positively charged −NH_2_ groups and many binding sites on the cell surface.

We demonstrate that the morphology of bacterial cells plays an important role in LV–carrier adhesion. The Gram-positive bacteria with a smooth surface adsorb less LV–carrier than Gram-negative bacteria *E. coli* with a rough surface and protein fimbria. Nevertheless, even without fimbria, the outer membrane of the Gram-negative bacteria prefers the absorbance of LV-CD than LV. Comparing LV-CDs and LV-CDpols, polymers are adsorbed 15% more for *E. coli*, in 2.3-fold for lactobacilli and 5-fold for *B. subtilis*.

CD and CDpol lowers MIC_LV_ by almost 2-fold. The TEM approach proves that CD carriers are not lethal for bacteria, but mild defects may occur that cannot be detected. We consider that CDs and CDpols might extract lipids and/or proteins from the bacterial surface that might cause mild damage in the membrane and, consequently, lead to the increase in LV’s antibacterial action.

## Figures and Tables

**Table 1:** The size and ζ-potential of CD, CDpol and their complexes with liposomes, pH 7.4 (0.02 M Na-phosphate buffer solution), 22 °C.

	Size, nm	ζ-Potential, mV	Mr, kDa	Number of CDs per 1 Particle	ζ-Potential of Liposomes + CD, mV
HPCD	0.15 ^1^	0.5 ± 0.1	1.5	−	−18.2 ± 4.4
HPCDpol	110 ± 15	9.2 ± 1.2	100 ± 15	67 ± 11	−14.3 ± 3.3
MCD	0.15 ^1^	0.7 ± 0.2	1.2	−	−19.6 ± 3.4
MCDpol	165 ± 15	22.9 ± 1.2	130 ± 15	100 ± 15	−12.2 ± 2.3
SBECD	0.15 ^1^	−7.7 ± 0.5	2.1	−	−24.8 ± 3.9
SBECDpol	110 ± 15	−13.4 ± 0.9	115 ± 13	65 ± 8	−14.2 ± 3.3
AMCD	0.15 ^1^	6.2 ± 1.2	1.6	−	−18.5 ± 3.6
AMCDpol	122 ± 17	10.2 ± 2.2	122 ± 15	70 ± 11	−13.5 ± 2.2
Liposomes DPPC:Cl = 80:20 (weight %)	105 ± 7	−20.9 ± 3.2	−	−	−

^1^ The size of CD derivatives is assumed to be close to the size of the parent CD [26].

**Table 2:** MIC values (μg/mL) for LV formulations, agar well diffusion method, pH 7.4 (0.02 M Na-phosphate buffer solution), 37 °C, 24 h of incubation.

	*E.coli* MH1	*E. coli* JM109	*E. coli* ATCC 25922	*L. fermentum* 90T-C4	*B. subtilis* ATCC 6633
LV	0.1 ± 0.02	1 ± 0.1	0.1 ± 0.02	45 ± 3	0.2 ± 0.03
MCD	-	-	-	-	-
AMCD	-	-	-	-	-
HPCDpol	-	-	-	-	-
MCDpol	-	-	-	-	-
LV-HPCD	0.12 ± 0.02	0.8 ± 0.2	0.05 ± 0.01	22 ± 3	0.15 ± 0.02
LV-MCD	0.1 ± 0.02	0.8 ± 0.2	0.05 ± 0.01	22 ± 2	0.14 ± 0.01
LV-SBECD	0.08 ± 0.02	1 ± 0.1	0.07 ± 0.02	20 ± 2	0.15 ± 0.02
LV-AMCD	0.08 ± 0.02	1.1 ± 0.2	0.07 ± 0.02	25 ± 3	0.13 ± 0.03
LV-HPCDpol	0.1 ± 0.02	1 ± 0.1	0.1 ± 0.02	45 ± 3	0.18 ±0.02
LV-MCDpol	0.13 ± 0.02	1 ± 0.1	0.1 ± 0.02	45 ± 3	0.15 ± 0.02
LV-SBECDpol	0.1 ± 0.02	0.9 ± 0.1	0.1 ± 0.02	45 ± 2	0.13 ± 0.03
LV-AMCDpol	0.12 ± 0.02	1 ± 0.2	0.1 ± 0.02	36 ± 3	0.15 ± 0.03

**Table 3:** ζ-potential (mV) of bacterial cells, pH 7.4 (0.02 M Na-phosphate buffer solution), 22 °C.

*E. coli* MH1	*E. coli* JM109	*E. coli* ATCC 25922	*L. fermentum* 90T-C4	*B. subtilis* ATCC 6633
−36.8 ± 4.5	−35.5 ± 5.6	−34.7 ± 4.8	−11.2 ± 0.5	−23.1 ± 0.5

## Data Availability

Not applicable.

## Supplementary Materials

The following supporting information can be downloaded at: https://www.mdpi.com/article/10.3390/polym14214476/s1, Figure S1: ^1^H NMR and FTIR spectra of MCD and MCDpol; Table S1: The slopes of adsorption curves.

## Abbreviations

The following abbreviations are used in this manuscript:

CD	Cyclodextrin
CDpol	Polymer based on cyclodextrin
HPCD	2-hydroxypropyl-β-cyclodextrin
MCD	Methyl-β-cyclodextrin
SBECD	Sulfobutyl ether β-cyclodextrin
AMCD	Amino-β-cyclodextrin
LV	Levofloxacin
CL	Cardiolipin
DPPC	Dipalmitoylphosphatidylcholine
PP	Phenolphthalein
MIC	Minimum inhibition concentration
CFU	Colony-forming units
FTIR	Fourier transform infrared spectroscopy
TEM	Transmission electron microscopes
UV	Ultraviolet spectroscopy
NTA	Nanoparticle data analysis
DLS	Dynamic light scattering
PXRD	Powder X-ray diffraction
