# Supplementary material for: Cyclodextrins and Their Polymers Affect the Lipid Membrane Permeability and Increase Levofloxacin’s Antibacterial Activity In Vitro
Source: Polymers (Basel). 2022 Oct 22;14(21):4476. doi: 10.3390/polym14214476 (PMC9654586; doi:10.3390/polym14214476)
Supplement: Supplementary file 1 [file polymers-14-04476-s001.zip › polymers-1922048-supplementary.pdf]

SUPPLEMENTARY

Figure S1. Normalized FTIR spectra of MCD and MCDpol (A), H_2O, 22°C. ^1H NMR spectra of MCD (B) and MCDpol (C), D_2O, 400 MHz.

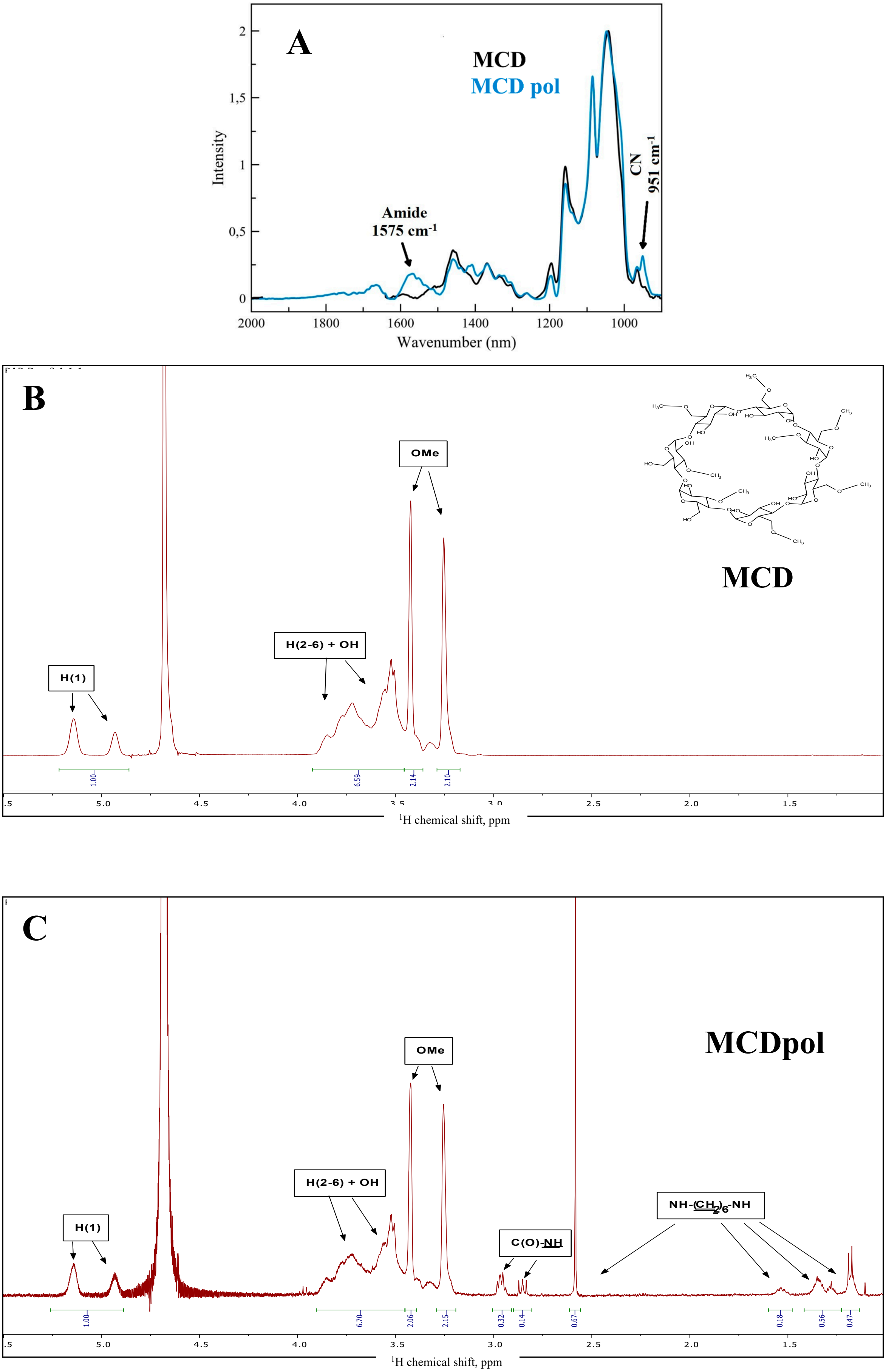

Table S1. The slopes of linear adsorption curves of LV and LV-CDs on *E. coli* strains.

	LV	LV-MCD	LV-HPCD	LV-SBECD
E. coli **MH 1**	0.20	0.49	0.34	0.29
E. coli **JM 109**	0.26	0.39	0.73	0.44
E. coli **ATCC 25922**	0.55	0.60	0.57	0.94
